# Interaction between valence of empathy and familiarity: is it difficult to empathize with the positive events of a stranger?

**DOI:** 10.1186/s40101-015-0049-3

**Published:** 2015-03-22

**Authors:** Yuki Motomura, Akira Takeshita, Yuka Egashira, Takayuki Nishimura, Yeon-kyu Kim, Shigeki Watanuki

**Affiliations:** Department of Psychophysiology, National Institute of Mental Health, National Center of Neurology and Psychiatry, 4-1-1 Ogawa-Higashi, Kodaira, Tokyo, 187-8553 Japan; Japan Society for the Promotion of Science, 5-3-1 Kojimachi, Chiyoda-ku, Tokyo, 102-0082 Japan; Integrative Brain Imaging Center, National Center of Neurology and Psychiatry, 4-1-1 Ogawa-Higashi, Kodaira, Tokyo, 187-8553 Japan; Faculty of Design, Kyushu University, 4-9-1 Shiobaru, Minami-ku, Fukuoka, 815-8540 Japan; Graduate School of Integrated Frontier Science, Kyushu University, 6-10-1 Hakozaki, Higashi-ku, Fukuoka, 812-8581 Japan; Department of Public Health, Nagasaki University Graduate School of Biomedical Sciences, Nagasaki, Japan

**Keywords:** Empathy, Event-related potential, Global field power, Friendship, Familiarity, Stranger, Friend

## Abstract

**Background:**

Empathy in humans is thought to have evolved via social interactions caused by the formation of social groups. Considering the role of empathy within a social group, there might be a difference between emotional empathy for strangers and familiar others belonging to the same social group. In this study, we used the global field power (GFP) index to investigate empathic brain activity during observation of a cue indicating either a negative or positive image viewed by a stranger or close friend.

**Methods:**

Sixteen healthy participants observed a partner performing an emotional gambling task displayed on a monitor. After the partner's choice-response, a frowning or smiling face symbol was simultaneously presented to the participant’s monitor while a negative or positive emotional image was presented to the partner’s monitor. All participants observed a control condition (CT) showing a computer trial, a stranger-observation condition (SO) showing the trial of a stranger, and a friend-observation condition (FO) to observe the trial of a close friend. During these observations, participants’ event-related potentials (ERPs) were recorded to calculate GFP, and after the task, a subjective assessment of their feelings was measured.

**Results:**

Positive emotion was significantly larger under the FO compared to the CT and the SO. Significantly larger negative emotion was found under the SO and FO compared to the CT. In response to a positive cue, significantly larger GFP during 300 to 600 ms was observed under the FO compared to the CT and SO. In response to a negative cue, significantly larger GFP was observed under the FO and SO compared to the CT. A significantly larger GFP under the SO was found in response to only a negative cue. Topographic map analysis suggested that these differences were related to frontal-occipital dynamics. GFP was significantly correlated with empathic trait.

**Conclusion:**

These results revealed that familiarity with another person has different effects depending on the valence of empathy. Negative empathy, including the danger perception function, might easily occur even among strangers, whereas positive empathy related to nursing and supporting an inner group does not happen easily with strangers.

## Background

Emotional empathy is defined as the same or related emotional experience caused by observing another’s emotional appearance [1]. It allows human beings to rapidly and automatically understand another’s emotion, and it facilitates successful social interactions. Empathy is reported to have not only short-term benefits such as enhanced social support, cooperation, and understanding but also long-term benefits such as enhanced friendship, reciprocity, and self-interest [2-4]. Individual empathic traits have a genetic basis [5], and infants also show empathic-like behavior [6,7]; thus, this ability is considered an innate and primitive mental function. Empathy is thought to have developed over the course of human evolution through social interactions caused by the formation of social groups. In ancient times, empathy with another’s anxiety or fear is thought to have played an important role in quickly recognizing survival-danger cues. On the other hand, pleasantness or sympathy caused by crying or smiling by others enhanced nursing and supportive behaviors within a social group, and consequently, these emotions might have enhanced individual survival rates [1,8].

Considering the role of empathy in a social group, emotional empathy for strangers and familiar others belonging to the same social group might differ. In fact, a previous study suggested that familiarity with an empathizer influenced empathy. To date, some studies using event-related potentials (ERP) recorded by electroencephalography or using functional magnetic resonance imaging (fMRI) have reported that empathic brain responses differ between strangers and familiar friends. Leng and Zhou showed enhanced amplitude of P300, which is an ERP component reflecting increased motivation and attention [9], in response to a friend’s financial loss or gain compared to that of a stranger’s while observing their performance during a gambling task [10]. Furthermore, they also compared another ERP component, feedback-related negativity (FRN), which is elicited during observation of one’s own loss or another’s loss compared to gain [9,11], but they found no significant difference between strangers and friends. A later study also found enhanced P300 amplitude during observation of a friend’s gain and loss compared to that of a stranger’s as found by Leng and Zhou, but they also found a larger FRN in response to a friend’s results than a stranger’s only when the empathizer did not concentrate strongly during the task [12]. In one fMRI study, brain response was measured while observing a friend or stranger’s exclusion in a task simulating social exclusion (Cyberball task) and found that observing a friend’s exclusion produced greater activation in the dorsal anterior cingulate cortex (ACC) and anterior insula, which are regions activated while observing another’s physical pain [13]. Moreover, activation correlated with self-reported psychological self-other overlap with a friend [14]. These studies consistently suggest that empathic brain responses elicited by another’s negative event is modulated by familiarity with the empathizer.

Whereas empathy for another’s negative event is known to be affected by familiarity, few reports have focused on the relationship between familiarity and empathy with positive emotion. However, each kind of empathy might show different characteristics for familiarity (e.g., empathy for negative emotion, including the function of danger perception, occurs more easily than for positive emotion). To our knowledge, no study has measured how the valence of emotional empathy (positive or negative) affects modulation of familiarity with the empathizer using a psychophysiological index such as the ERP. In this study, we used an index called the global field power (GFP) [15], which is the standard deviation of the electric field at the scalp, and is thought to reflect neuronal dynamics throughout the brain. In conventional ERP analyses, although undoubtedly informative, the peak analysis of empirically defined ERP components has some pitfalls such as bias in analysis, changes in amplitude dependent on reference electrodes, and risk of missing a potentially important modulation of low-amplitude waveforms [16,17]. GFP can avoid these pitfalls in conventional ERP analysis [15,17,18]. Furthermore, empathy is thought to be an integrative higher-grade function that includes various cognitive and emotional processes such as understanding of another person’s situation, affective imagination of another person, and expression of one’s emotions [1]. Neuroimaging studies have shown an association between empathy and various neural networks including the amygdala, anterior insula, ACC, precuneus, and frontal orbital cortex [19-21]. Such electroencephalogram (EEG) brain activities are thought to delocalize all over the scalp. GFP, as an interactive index throughout the brain, can therefore be more appropriate for capturing higher-grade functions such as empathy than local brain potentials. In fact, GFP can capture differential brain characteristics in face recognition [22], semantic learning of kanji characters [23], and responses to multisensory stimuli [24]. Therefore, we used subjective assessment and global brain activity to investigate empathic response during observation of cues indicating either a negative or positive image viewed by a stranger or close friend.

## Material and methods

### Ethics

This study was approved by the Ethics Committee of Kyushu University and was conducted in accordance with the Declaration of Helsinki.

### Participants

Participants were 16 healthy, right-handed university students (mean age ± standard deviation, 24.1 ± 3.32 years; 8 men, 8 women) who provided written informed consent prior to participating in the study. Participants were asked to get adequate sleep and refrain from alcohol and hard exercise the day before the experiment and to refrain from caffeine intake and smoking 1 h before the experiment.

### Experimental protocol

**Figure 1:**
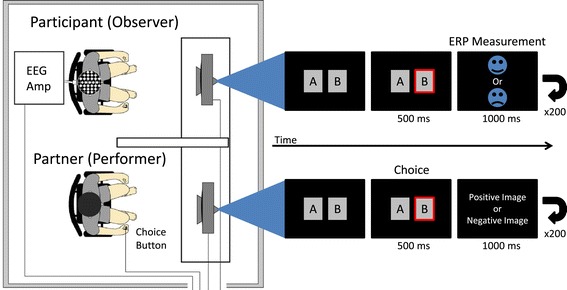
**Emotional gambling task design.** Participants observed their partner performing an emotional gambling task through a monitor displaying the task. Two boxes, labeled A and B (angle of field, 2.62° × 3.59°; interval between boxes, 2.76°), were displayed on each of their monitors. Each time their partner presses a button to choose either A or B, an emotional negative or positive image is presented 500 ms after the choice on only the partner’s monitor and simultaneously a sad or happy face symbol is presented on the participant’s monitor. Each valence (positive or negative) trial was performed 100 times, respectively, and a total of 200 images or face symbols were presented. EEG Amp = electroencephalogram amplifier; ERP = event-related potentials.

Each participant observed his or her partner performing an emotional gambling task (Figure 1) that was based on a monetary gambling task [25] and modified to elicit emotional empathy. The monitors of both the participant and the partner displayed the same gambling task. Each participant visited the lab a total of three times to participate in a control condition (CT), in which a computer trial was observed with no partner seated next to the participant; a stranger-observation condition (SO), in which the trial of a partner the participant was seeing for the first time; and a friend-observation condition (FO), in which the trial of a close friend whom the participant had had known for over 2 years was observed. The order of each condition was counterbalanced, and conditions were tested at intervals of more than 2 days, with all conditions carried out within 1 month.

Figure 1 shows the emotional gambling task protocol. First, two boxes labeled A and B (angle of field, 2.62° × 3.59°; interval between boxes, 2.76°) were displayed on a monitor. The partner chose either box A or B by pressing a button. Both participant and partner were informed beforehand that if the partner chose the wrong box, an image eliciting a negative emotion such as rotten food, insects, internal organs, or dead animals would be shown, but if the right box was chosen, an image eliciting a positive emotion such as a beautiful flower, magnificent landscape, or beautiful building would be shown for 1,000 ms on only the partner’s monitor (positive and negative images were actually presented an equal number of times in random order). Simultaneously, a frowning or smiling face symbol was displayed on the participant’s monitor and a negative or positive image was displayed on the partner’s monitor. A trial for each valence (positive or negative face symbols) was performed 100 times, and 200 images or face symbols were presented in total. Brain waves of the participants (observers) during presentation of the face symbol were averaged, and time-locked ERPs were acquired. Participants and their partners were prohibited from conversing throughout the experiment. The task program script was coded using Windows Visual Basic 6.0, and stimuli were presented using Multi Trigger System (MTS0410, Medical Try System, Tokyo, Japan) triggered by the task program. The monitor refresh rate was set to 75 Hz during task presentation, and the response speed was 5 ms (LCD-A 173KB-X, I/O DATA, Tokyo, Japan).

### Questionnaires

Participants came to the lab to answer questionnaires. We assessed the participants’ anxiety and empathic traits using the trait components of the State-Trait Anxiety Inventory [26] (STAI-T) and a Multidimensional Empathy Scale for Adolescents (MESA) [27] (a Japanese questionnaire based on the Interpersonal Reactivity Index [28]). For all conditions, participants answered the state component of the STAI [26] (STAI-S). In the stranger and friend conditions, to measure self-other overlap with the experiment partner, participants answered the Inclusion of Other in the Self (IOS) Scale [29]. Participants chose the item that best expressed the relationship between their self and their experiment partner among 1 to 7 items, which showed two gradually overlapping circles. Two participants (one man and one woman) who had chosen item 1, the most spread apart circles, to describe a close friend under the FO were excluded from all analyses (questionnaire, subjective assessment, and EEG). The participants also reported the duration from the time when they first met their experiment partner.

### Subjective assessment

After each task, the participants answered the following subjective assessment questions regarding how they felt during the task: you concentrated on the task (concentration); you were interested in the task (interest); when a result was displayed, you paid attention to your partner’s result (attention); you felt your emotion changes as a result of your partner’s result (emotional movement); you imagined how you would feel if you were presented with those emotional images (perspective taking); when you observed a positive result for your partner, you felt glad, relieved, or that your partner was fortunate (positive emotions); and when you observed a negative result for your partner, you felt sorrow, pain, a thumping heart, sympathy, shaken, or confused (negative emotions).

For each question, the participants determined how closely the questions matched their own feelings using numbers from 1 (*match*) to 9 (*does not match*). For clarity of presentation, each value was inverted to a larger number showing greater matching of subjective state (replace 1 with 9, 2 with 8, 3 with 7, and 4 with 6, respectively). A positive emotions and negative emotions score was averaged using scores of each item. Due to data loss for the question on positive emotions, we excluded three participants’ responses from the analysis of this question.

### Event-related potential measurement and analysis

EEG was acquired using a 64-channel net (HydroCel Geodesic Sensor Net, Electrical Geodesics, Inc., Eugene, OR, USA) and amplified and measured (Net Amps 200 64-channel EEG Amplifier, Electrical Geodesics, Inc., Eugene, OR, USA; Net Station version 4.1.2, Electrical Geodesics, Inc., Eugene, OR, USA). Electrode resistance was maintained at ≤100 kΩ during the experiment, and data were continuously recorded at a sampling frequency of 250 Hz and electrode on Cz was used as the system reference. The hardware band-pass filter was set at 0.1 to 100 Hz. EMSE data editor version 5.2 was used for analysis. Measures of EEGs were transformed using electrodes on mastoids as the offline reference, and a software band-pass filter (0.1 to 30 Hz) was applied. Trials including artifacts above ±40 μV were rejected manually. Face symbol presentation for participants was set as 0 ms, and a −200- to 800-ms range was averaged to obtain an ERP waveform in each of the six conditions: CT-positive, CT-negative, SO-positive, SO-negative, FO-positive, and FO-negative. Baseline correction of ERP was carried out by subtracting the mean value of −200 to 0 ms from the overall waveform. The number of additions to average was set at ≥60 times.

### Global field power analysis

GFP is defined as the standard deviation of electrical potentials across all EEG electrodes at a given time [15]. GFP is thought to reflect neuronal dynamics throughout the brain. We computed the mean value of GFP in the time window from 0 to 600 ms post-stimulus, which is an obvious component shown in the grand-mean GFP waveform. We generated topographic maps using all participants and all conditions from the grand-mean ERP data with time courses. Next, because GFP fluctuated with the overlapping components of all electrodes averaged in the grand-mean ERP, we divided GFP into four time windows corresponding to N1 (80 to 150 ms), P2 (150 to 220 ms), N2 (220 to 300 ms), and P3 (300 to 600 ms) of the ERP and analyzed it in each time window (N1-GFP, P2-GFP, N2-GFP, P3-GFP). Using ERP data, we generated topographic maps for each of the six conditions and for each component.

### Correlation analysis with empathic trait

We evaluated the correlation of individual empathic traits (MESA subscales: empathic concern, personal distress, fantasy, and perspective taking) with the P3-GFP value, which shows the main effect of the observation condition. Because inter-individual variance in P3-GFP value was much larger than intra-individual variance (intra-class class correlation 0.98, *P* < 0.001), we considered the Δ value, which is the difference of each individual CT value in SO and FO for each valence (SO-positive, SO-negative, FO-positive, FO-negative).

### Statistics

SPSS PASW Statistics 18 software was used for statistical analysis. The Friedman test with presentation conditions (CT, SO, or FO) as factors was conducted for behavioral data. When a significant difference was found, we applied the Wilcoxon signed-rank test for *post hoc* tests with Bonferroni step-down (Holm) correction for multiple comparisons. For EEG data, a two-way repeated measures ANOVA was conducted with face symbol (positive or negative) valence and presentation conditions (CT, SO, or FO) as factors. When a significant main effect or interaction was found, we applied a *post hoc* paired *t*-test with Holm correction for multiple comparisons (*post hoc t*-test was performed nine times for EEG analysis (positive *vs*. negative in each presentation condition and CT *vs*. SO, SO *vs*. FO, FO *vs*. CT for each valence), a significance level of 0.05 * 1/9 ~ 1/1 was applied). For clarity, the *P*-values are shown as a multiplied value corresponding to each Holm correction threshold. When sphericity was not assumed, we used the value after Greenhouse-Geisser correction. All data were considered significant at *P* < 0.05.

## Results

### Demographic data

Table 1 shows participants’ age, sex ratio, anxiety traits, and empathic traits.

**Table 1:** **Demographic data**

	**Mean (±SD)**
Age	22 (1.47)
Male:female	1:1
STAI-Trait	31.5 (8.11)
MESA empathic concern	3.72 (0.34)
MESA personal distress	3.33 (0.84)
MESA fantasy	3.48 (1.04)
MESA perspective taking	3.06 (0.57)

*Note.* Number of participants = 14. SD = standard deviation; STAI = State-Trait Anxiety Inventory; MESA = Multidimensional Empathy Scale for Adolescents.

### Subjective assessment

**Figure 2:**
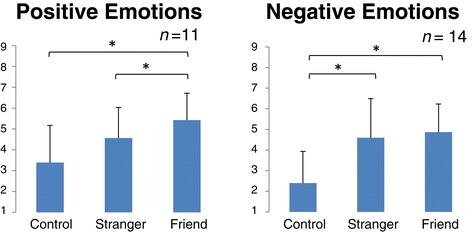
**Subjective assessment score of positive and negative emotions.** Positive emotions was the average of glad, relieved, and feeling the partner was fortunate, and negative emotions was the average of feeling sorry, pain, a thumping heart, sympathy, shaken, and confused. Higher scores signify a higher match with the participant’s own feeling. The graph shows the mean value and standard error. Degrees of freedom (*df*) = 10, 13, respectively. *P < 0.05 by Holm correction.

Table 2 shows the results of partner familiarity and subjective assessment after observation of the partner’s performance during the emotional gambling task. A significant main effect in the observation condition for perspective taking (*F* [2] = 6.30, *P* < 0.05) after *post hoc* tests was found to be marginally significant between the CT and the SO or FO. By the Friedman test, a significant effect in the observation condition was found for positive emotions (*F* [2] = 8.14 *P* < 0.05) and negative emotions (*F* [2] = 16.72, *P* < 0.001). *Post hoc* tests revealed a higher positive emotions score in the FO compared to the CT and SO. We found a higher score for negative emotions in the SO and FO compared to the CT (Figure 2). No significant effects were found in STAI-S, concentration, interest, attention, or emotional movement (*F* [2] = 0.44, *P* = 0.8; *F* [2] = 3.82, *P* = 0.15; *F* [2] = 1.02, *P* = 0.60; *F* [2] = 2.18, *P* = 0.34; and *F* [2] = 3.29, *P* = 0.19, respectively).

**Table 2:** **Subjective assessment scores**

	**Control (CT)**	**Stranger observation (SO)**	**Friend observation (FO)**	***P*** **value (Friedman test)**	***Post hoc*** **(Wilcoxon signed-rank test)**
Duration		0 (0)	42.6 (13)		SO < FO (*P* < 0.001)
IOS Scale		1.5 (0.65)	5.29 (1.2)		SO < FO (*P* < 0.001)
STAI-State	41.43 (8.02)	42.42 (7.68)	41.85 (10.66)	ns	
Concentration	6.93 (1.64)	6.14 (2.41)	7.29 (1.51)	ns	
Interest	5.86 (1.92)	6.07 (1.59)	6.36 (1.45)	ns	
Attention	6.14 (2.35)	6.21 (2.39)	6.93 (1.69)	ns	
Emotional movement	3.64 (2.06)	4.21 (2.12)	6 (1.92)	ns	
Perspective taking	3 (1.88)	4.64 (2.1)	4.86 (2.54)	<0.05.	CT < SO (*P* < 0.1)
					CT < FO (*P* < 0.1)
Negative emotions	2.4 (1.54)	4.59 (1.9)	4.87 (1.36)	<0.001	CT < SO (*P* < 0.05)
					CT < FO (*P* < 0.01)
Positive emotions	3.39 (1.78)	4.56 (1.47)	5.43 (1.28)	<0.05	CT < FO (*P* < 0.05)
					SO < FO (*P* < 0.05)

*Note.* All values are expressed as mean (standard deviation). Degrees of freedom (*df*) = 13, only positive emotions (*df*) = 10. *Post hoc* test was performed with Holm correction. CT = control condition; SO = stranger-observation condition; FO = friend-observation condition; STAI = State-Trait Anxiety Inventory; IOS = Inclusion of Other in the Self Scale; ns = not significant.

### ERP data

**Figure 3:**
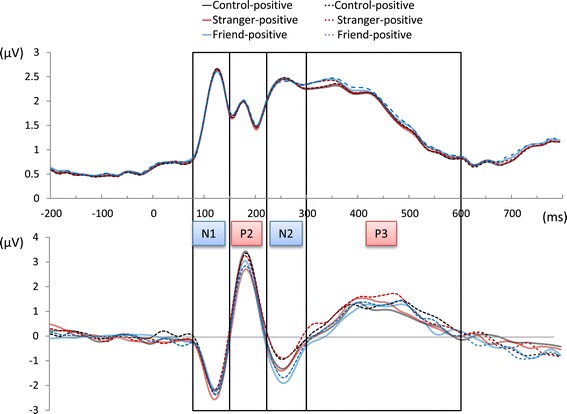
**GFP and grand-mean ERP waveform.** The upper graph shows the GFP waveform, and under the graph are all electrodes averaged in the grand-mean ERP waveform. GFP fluctuated with the overlapping components of the grand-mean ERP. We performed GFP analysis by dividing GFP into four time windows: N1 (80 to 150 ms), P2 (150 to 220 ms), N2 (220 to 300 ms), and P3 (300 to 600 ms). GFP = global field power; ERP = event-related potential.

Figure 3 shows the GFP waveform during observation of face symbols with all electrodes averaged in the grand-mean ERP. GFP fluctuated with the overlapping components of the ERP.

**Figure 4:**
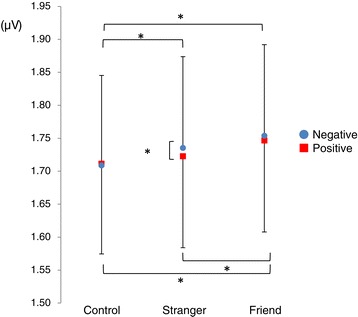
**Global field power of P3.** Mean value of P3-GFP (300 to 600 ms) under each condition. Red and blue points show GFP during positive and negative cues. The graph shows the mean value and standard error. Degrees of freedom (*df*) = 13. *P < 0.05 by Holm correction.

We found a significant main effect of valence (*F* [1,13] = 4.82, *P* < 0.05) in N1-GFP. We did not find a significant main effect of the observation condition or interaction between valence and the observation condition (*F* [2,26] = 1.67, *P* = 0.22 and *F* [2,26] = 1.34, *P* = 0.28, respectively). Results of *post hoc* tests revealed a significantly larger GFP in response to negative cues than positive cues under the CT. No significant main effect and interaction were seen in P2-GFP (valence effect, *F* [1,13] = 2.18, *P* = 0.16; observation condition effect, *F* [2,26] = 2.11, *P* = 0.17; interaction, *F* [2,26] = 0.41, *P* = 0.61) and N2-GFP (valence effect, *F* [1,13] = 1.00, *P* = 0.34; observation condition effect, *F* [2,26] = 1.33, *P* = 0.28; interaction, *F* [2,26] = 0.18, *P* = 0.84). We found a significant main effect under observation conditions (*F* [2,26] = 11.18, *P* < 0.01), and their interaction (*F* [2,26] = 4.24, *P* < 0.05) in P3-GFP (Figure 4), and a significant main effect in valence was not found (*F* [1,13] = 2.70, *P* = 0.12). *Post hoc* tests revealed a significantly larger GFP in response to a positive cue under the FO compared to both the CT and the SO. On the other hand, in response to a negative cue under the SO, a significantly larger GFP was found compared to the CT. Significantly larger GFP was also found under the FO compared to the CT. Furthermore, under only the SO was a larger GFP found in response to a negative cue than a positive cue.

**Figure 5:**
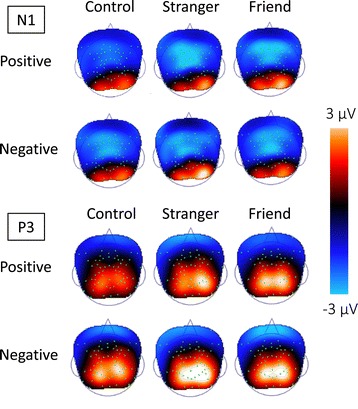
**N1 and P3 topographic maps.** The upper map shows the N1 (80 to 150 ms) map under each condition, and the lower map shows the P3 (300 to 600 ms) map. Red and blue areas show high amplitude of positive and negative potentials.

Figure 5 shows topographic maps of N1 and P3. In all conditions in N1, we observed frontal- and parietal-dominant negative potentials and occipital-dominant negative potentials. In P3, we observed frontal-dominant negative potentials and occipital- and parietal-dominant negative potentials. GFP reflects the amplitude of the contrast among these local potentials of differing electrical polarity.

### Correlation with empathic trait

Table 3 shows a correlation matrix between empathic trait and P3-GFP for each condition. Fantasy score was positively correlated with P3-GFP in all conditions (SO-positive, *r* [14] = 0.78, *P* < 0.01; SO-negative, *r* [14] = 0.70, *P* < 0.01; FO-positive, *r* [14] = 0.73, *P* < 0.01; FO-negative, *r* [14] = 0.64, *P* < 0.01). Personal distress score was positively correlated with P3-GFP in the SO-positive condition and SO-negative condition (*r* [14] = 0.66, *P* < 0.05; *r* [14] = 0.62, *P* < 0.05) and marginally significant in the FO-positive condition (*r* [14] = 0.48, *P* = 0.081).

**Table 3:** **Correlation between empathic trait and P3-GFP**

	**Empathic concern**	**Personal distress**	**Fantasy**	**Perspective taking**
SO-positive (minus CT-positive)	0.30	0.66*	0.78**	0.11
SO-negative (minus CT-negative)	0.19	0.62*	0.70**	−0.09
FO-positive (minus CT-positive)	0.08	0.48†	0.73**	−0.01
FO-negative (minus CT-negative)	−0.02	0.36	0.64*	−0.20

*Note.* CT = control condition; SO = stranger-observation condition; FO = friend-observation. †*p* < 0.1; *p < 0.05; **p < 0.01.

## Discussion

The results of this study revealed that familiarity with an empathizer has different effects depending on the valence of empathy (negative or positive). Empathy elicited by the negative event of either a stranger or close friend was larger than the control. Also, empathy elicited by the positive event of a friend was larger than that elicited by the control, whereas the empathic response to a stranger was not larger than that to the control (Table 2, Figure 2). These results are supported by EEG data and reveal that empathy for another’s negative emotions occurs easily regardless of familiarity, whereas empathy for positive emotions of a stranger does not occur as easily as it does for a close friend (Figure 4). EEG results reflect emotional empathy for partners because we found no significant effect for anxiety, concentration, interest, or attention during the task, but did find a significant effect for subjective positive and negative emotions that corresponded to EEG data (Table 2).

Time-window analysis showed a significant main effect of valence in N1-GFP. In this time course, N1 reportedly related to lower processes such as bottom-up attention [30] and perceived brightness of visual stimuli [31]. It can reflect a difference in visual characteristics between positive and negative valences by different image presentations (happy face symbol or sad face symbol) in each task trial.

**Figure 6:**
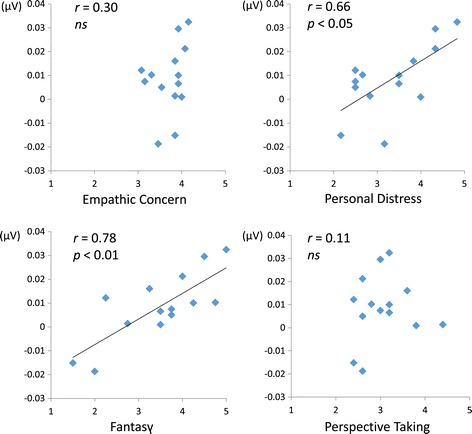
**Scatter plots for empathic traits and P3-GFP in SO-positive condition.** Vertical axis = amplitude of P3-GFP; horizontal axis = subscale score of Multidimensional Empathy Scale for Adolescents.

We also found a significant main effect for the observation condition and their interaction in P3-GFP (Figure 4). This finding suggests that the influence of familiarity on emotional empathic brain activity is strongly related to late (after 300 ms) processes rather than early processes. Moreover, from the topographic map of this time course, P3-GFP is supposed to reflect the contrast between negative potentials of the frontal region and positive potentials of the occipital region (Figure 5). These results suggest that empathy is influenced by familiarity with others via modulation of frontal-occipital dynamics of late neural processes occurring after 300 ms. Furthermore, the correlation of P3-GFP and some of the empathic traits supported that the index could accurately reflect empathic brain activity (Table 3, Figure 6). In all conditions, the Fantasy subscale significantly correlated with the amplitude of P3-GFP. Individuals with a higher Fantasy score easily empathize with fictional characters in novels or movies. The Fantasy subscale is associated with not only internal empathic feeling but also external empathic responses such as social anxiety, shyness, and feeling isolated [32,33]. In this study, in addition to such strong empathic responses, it is inferred that strong self-projection to the experimental task (as well as immersing themselves in a story) caused strong empathic brain responses regardless of familiarity. Attention should be paid to the fact that a significant correlation with the personal distress score was observed in the SO-positive condition, which did not show a significant difference in P3-GFP in comparison with the CT. Individuals with a higher personal distress score easily feel a personally oriented distress response to another individual’s negative emotions [33]. They can even be more easily affected by another individual’s positive emotions as well.

We can interpret these results as follows. Negative empathy, including the danger perception function, was elicited easily regardless of one’s familiarity with another person. On the other hand, positive emotion, which was associated with nursing or supporting behaviors in ancient times, is difficult to feel for strangers who do not belong to one’s group. Previous studies have shown that empathy is influenced by social context or the empathizer’s emotional condition. For example, Singer and colleagues showed that empathy for another’s physical pain was affected by the social attitudes of other participants [34]. Male participants had a different emotional empathic response to a person who exhibited betraying behavior. They showed brain responses to a trustworthy person in regions such as the ACC and anterior insula, which is related to emotional empathy [19]. In contrast, brain response to physical pain caused by betrayers was observed in the nucleus accumbens, which is reportedly related to the rewarding system. In our study, although strangers were neutrally related to the participants, we expected the inverse valence of empathy, such as envy and *schadenfreude* (feeling happy about another’s misery), would be shown for opposing strangers corresponding to an invader of the group.

## Conclusion

The results of this study revealed that familiarity with another person has different effects depending on the valence of empathy. Negative empathy that includes the danger perception function possibly occurs easily even with strangers, whereas positive empathy related to nursing and supporting the inner group does not. Because social interaction between or within groups is thought to be one of the selective pressures that shaped brain evolution as a result of group formation [35], we expect that further investigation of the influence of familiarity on social brain function can help clarify the evolution of the human brain.

## Abbreviations

ACC: anterior cingulate cortex
CT: control condition
EEG: electroencephalogram
ERP: event-related potential
fMRI: functional magnetic resonance imaging
FO: friend-observation condition
FRN: feedback-related negativity
GFP: global field power
SO: stranger-observation condition

## References

[1] Davis MH (1994). Empathy: a social psychological approach.

[2] Rumble AC, Van Lange PAM, Parks CD (2010). The benefits of empathy: when empathy may sustain cooperation in social dilemmas. Eur J Soc Psychol.

[3] Lakin JL, Chartrand TL (2003). Using nonconscious behavioral mimicry to create affiliation and rapport. Psychol Sci.

[4] Batson CD, Ahmad N (2001). Empathy-induced altruism in a prisoner’s dilemma II: what if the target of empathy has defected?. Eur J Soc Psychol.

[5] Knafo A, Zahn-Waxler C, Van Hulle C, Robinson JL, Rhee SH (2008). The developmental origins of a disposition toward empathy: genetic and environmental contributions. Emotion.

[6] Roth-Hanania R, Davidov M, Zahn-Waxler C (2011). Empathy development from 8 to 16 months: early signs of concern for others. Infant Behav Dev.

[7] Sagi A, Hoffman ML (1976). Empathic distress in the newborn. Dev Psychol.

[8] de Waal FB (2008). Putting the altruism back into altruism: the evolution of empathy. Annu Rev Psychol.

[9] Nieuwenhuis S, Aston-Jones G, Cohen JD (2005). Decision making, the P3, and the locus coeruleus-norepinephrine system. Psychol Bull.

[10] Leng Y, Zhou X (2010). Modulation of the brain activity in outcome evaluation by interpersonal relationship: an ERP study. Neuropsychologia.

[11] Gehring WJ, Willoughby AR (2002). The medial frontal cortex and the rapid processing of monetary gains and losses. Science.

[12] Ma Q, Shen Q, Xu Q, Li D, Shu L, Weber B (2011). Empathic responses to others’ gains and losses: an electrophysiological investigation. Neuroimage.

[13] Singer T, Seymour B, O’Doherty J, Kaube H, Dolan RJ, Frith CD (2004). Empathy for pain involves the affective but not sensory components of pain. Science.

[14] Meyer ML, Masten CL, Ma Y, Wang C, Shi Z, Eisenberger NI (2013). Empathy for the social suffering of friends and strangers recruits distinct patterns of brain activation. Soc Cogn Affect Neurosci.

[15] Lehmann D, Skrandies W (1980). Reference-free identification of components of checkerboard-evoked multichannel potential fields. Electroencephalogr Clin Neurophysiol.

[16] Pourtois G, Thut G, Grave De Peralta R, Michel C, Vuilleumier P (2005). Two electrophysiological stages of spatial orienting towards fearful faces: early temporo-parietal activation preceding gain control in extrastriate visual cortex. Neuroimage.

[17] Murray MM, Brunet D, Michel CM (2008). Topographic ERP analyses: a step-by-step tutorial review. Brain Topogr.

[18] Doi H, Shinohara K (2015). Unconscious presentation of fearful face modulates electrophysiological responses to emotional prosody. Cereb Cortex.

[19] Fan Y, Duncan NW, de Greck M, Northoff G (2011). Is there a core neural network in empathy? An fMRI based quantitative meta-analysis. Neurosci Biobehav R.

[20] Singer T (2006). The neuronal basis and ontogeny of empathy and mind reading: review of literature and implications for future research. Neurosci Biobehav R.

[21] Chakrabarti B, Bullmore ET, Baron-Cohen S (2006). Empathizing with basic emotions: common and discrete neural substrates. Soc Neurosci-UK.

[22] Bernasconi F, Schmidt A, Pokorny T, Kometer M, Seifritz E, Vollenweider FX (2014). Spatiotemporal brain dynamics of emotional face processing modulations induced by the serotonin 1A/2A receptor agonist psilocybin. Cereb Cortex.

[23] Shinoda H, Skrandies W (2013). Topographic changes in event-related potentials because of learning of meaningful Kanji characters. Neuroreport.

[24] Hiessl AK, Skrandies W (2013). Evaluation of multisensory stimuli–dimensions of meaning and electrical brain activity. Neuropsychologia.

[25] Yeung N, Holroyd CB, Cohen JD (2005). ERP correlates of feedback and reward processing in the presence and absence of response choice. Cereb Cortex.

[26] Spielberger CD, Gorssuch RL, Lushene PR, Vagg PR, Jacobs GA. Manual for the State-Trait Anxiety Inventory. Consulting Psychologists Press, Inc., 1983

[27] Tobari M (2003). The development of empathy in adolescence: a multidimensional view. Jpn J Dev Psychol.

[28] Davis MH (1980). A multidimensional approach to individual differences in empathy. JSAS Catalog of Selected Documents in Psychology.

[29] Woosnam KM (2010). The inclusion of other in the self (IOS) scale. Ann Tourism Res.

[30] Luck SJ, Heinze HJ, Mangun GR, Hillyard SA (1990). Visual event-related potentials index focused attention within bilateral stimulus arrays. II. Functional dissociation of P1 and N1 components. Electroencephalogr Clin Neurophysiol.

[31] Johannes S, Munte TF, Heinze HJ, Mangun GR (1995). Luminance and spatial attention effects on early visual processing. Brain Res Cogn Brain Res.

[32] Fontenelle LF, Soares ID, Miele F, Borges MC, Prazeres AM, Range BP (2009). Empathy and symptoms dimensions of patients with obsessive-compulsive disorder. J Psychiatr Res.

[33] Davis MH (1983). Measuring individual differences in empathy: evidence for a multidimensional approach. J Pers Soc Psychol.

[34] Singer T, Seymour B, O’Doherty JP, Stephan KE, Dolan RJ, Frith CD (2006). Empathic neural responses are modulated by the perceived fairness of others. Nature.

[35] Dunbar RI (2009). The social brain hypothesis and its implications for social evolution. Ann Hum Biol.

